# Identification of Parthenogenesis-Inducing Effector Proteins in *Wolbachia*

**DOI:** 10.1093/gbe/evae036

**Published:** 2024-03-26

**Authors:** Laura C Fricke, Amelia R I Lindsey

**Affiliations:** Department of Entomology, University of Minnesota, St. Paul, MN 55108, USA; Department of Entomology, University of Minnesota, St. Paul, MN 55108, USA

**Keywords:** symbiosis, *Wolbachia*, parthenogenesis, parasitoid, sex, mitosis

## Abstract

Bacteria in the genus *Wolbachia* have evolved numerous strategies to manipulate arthropod sex, including the conversion of would-be male offspring to asexually reproducing females. This so-called “parthenogenesis induction” phenotype can be found in a number of *Wolbachia* strains that infect arthropods with haplodiploid sex determination systems, including parasitoid wasps. Despite the discovery of microbe-mediated parthenogenesis more than 30 yr ago, the underlying genetic mechanisms have remained elusive. We used a suite of genomic, computational, and molecular tools to identify and characterize two proteins that are uniquely found in parthenogenesis-inducing *Wolbachia* and have strong signatures of host-associated bacterial effector proteins. These putative parthenogenesis-inducing proteins have structural homology to eukaryotic protein domains including nucleoporins, the key insect sex determining factor Transformer, and a eukaryotic-like serine–threonine kinase with leucine-rich repeats. Furthermore, these proteins significantly impact eukaryotic cell biology in the model *Saccharomyces cerevisiae*. We suggest that these proteins are parthenogenesis-inducing factors and our results indicate that this would be made possible by a novel mechanism of bacterial-host interaction.

**Significance**

Microbe-mediated asexual reproduction in arthropods was discovered more than 30 yr ago, but the underlying mechanisms have since remained elusive. Using a combination of comparative and functional genomics, we identified two genes from parthenogenesis-inducing *Wolbachia* symbionts that we predict mediate the transition of the host to asexual reproduction. This discovery is the first step toward understanding how these symbionts interact with host cell division and sex determination, ultimately causing major shifts in arthropod reproduction.

## Introduction

The evolution of eukaryotic organisms has been continuously sculpted by relationships with intracellular microbes. The characteristics of these intracellular organisms have been driven by strong selection pressures to manipulate host physiology in favor of their own transmission and persistence (Moran et al. 2008). Many endosymbionts can be transmitted maternally but not paternally, an asymmetry that can result in sexual and genetic conflicts (Perlman et al. 2015). This phenomenon is exemplified by one of the most prevalent endosymbionts on earth, the bacterium *Wolbachia*. *Wolbachia* are maternally transmitted alphaproteobacteria (Rickettsiales) in many filarial worms and arthropods, including around half of all insect species (Kaur et al. 2021). Most remarkably, *Wolbachia* can drastically alter host reproductive outcomes in favor of their own transmission and spread, including the conversion of would-be male offspring to *Wolbachia*-transmitting genetic females, i.e. the induction of parthenogenesis.

**Fig. 1.**
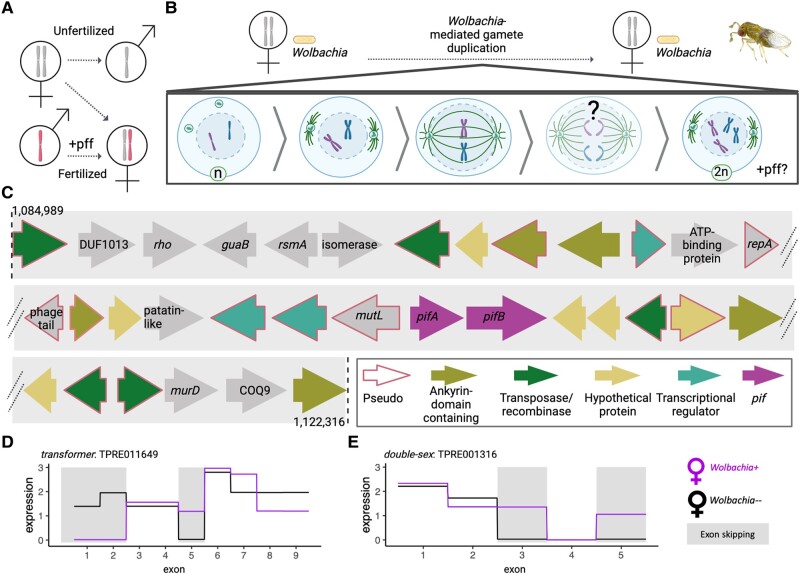
*Wolbachia-mediated parthenogenesis* and putative PI factors. A) Haplodiploid sex determination in the absence of *Wolbachia*. Haploid males develop from unfertilized eggs, and diploid females develop from fertilized eggs. In some sexual hymenopterans, a paternal factor is required for driving female development in the offspring on top of fertilization (+pff). B) Parthenogenesis-inducing *w*Tpre *Wolbachia* causes gamete duplication via a failed anaphase during the first mitotic division in *Trichogramma* embryos in the absence of fertilization. C) Candidate PI factors (*pifs*) are located within a degraded EAM. The schematic shows coding regions from base pairs 1,084,989 to 1,122,316 of the *w*Tpre reference (National Center for Biotechnology Information (NCBI) Reference Sequence: NZ_CM003641.1; Lindsey et al 2016). D) Exon use of *transformer* and E) *double-sex* in female *T. pretiosum* with and without *Wolbachia* infections. Exons that are differentially skipped between *Wolbachia*-infected and -uninfected wasps are indicated with gray boxes. Schematics created with BioRender.com. *Trichogramma* photo taken by A.R.I.L.

Parthenogenesis induction (PI) is arguably the most advantageous reproductive phenotype for *Wolbachia*, as it is the only known symbiont-mediated reproductive manipulation in which host populations can be sustained with females only, resulting in all hosts being avenues for transmission of *Wolbachia* to the next generation. To date, PI-*Wolbachia* strains have been confirmed in a range of haplodiploid arthropods, including parasitoid wasps, thrips, and mites (Ma and Schwander 2017). Haplodiploidy is a sex determination system that relies on ploidy of the embryo: in the simplest form, males are haploid, and females are diploid. In many lineages, this difference in ploidy derives from whether a mother fertilizes a given egg, thus restoring diploidy (Gietz and Woods 2002; Fig. 1A). However, in the presence of PI-*Wolbachia*, diploid females can be produced from unfertilized eggs through a fertilization-independent, *Wolbachia*-mediated restoration of diploidy (Fig. 1B).

PI has evolved multiple times across *Wolbachia* as well as in several other genera of arthropod endosymbionts (Ma and Schwander 2017). Different occurrences of microbe-mediated PI are hypothesized to be made possible through different mechanisms, as inferred by cytogenetic approaches combined with estimates of heterozygosity (Stouthamer and Kazmer 1994; Gottlieb and Zchori-Fein 2001; Weeks and Breeuwer 2001; Pannebakker et al. 2004; Ma and Schwander 2017). For example, some PI-*Wolbachia* target meiosis to generate diploid gametes, while others target mitosis to convert haploid embryos to diploid. The PI-*Wolbachia* strains *w*Tpre and *w*Lcla cause a failed anaphase in the first mitotic division of the embryo leading to a single diploid nucleus in the parasitoids *Trichogramma* and *Leptopilina*, respectively (Fig. 1B; Stouthamer and Kazmer 1994; Pannebakker et al. 2004), while the strain “*w*Uni” causes gamete duplication through a tetraploid second mitotic prophase in a different parasitoid genus (Gottlieb and Zchori-Fein 2001), and “*w*Bpra” induces functional apomixis in a species of mite (Weeks and Breeuwer 2001). Thus, PI bacteria have developed a variety of mechanisms for impacting host chromosome segregation.

In addition to differences in the timing and mechanisms for altering ploidy, there appears to be variation in whether a change in ploidy is sufficient to facilitate female development. In the *w*Uni and *w*Bpra systems, the diploidization event appears to be sufficient on its own to ensure female development. These have been termed “one-step PI” systems (Verhulst et al. 2023). In other PI systems, diploidization alone is not sufficient, as evidenced by the occasional presence of diploid phenotypic males, and even haploid and diploid individuals with intersex characteristics, especially when *Wolbachia* titers have been experimentally reduced (Tulgetske 2010; Lindsey and Stouthamer 2017). This implies a two-step mechanism of PI, wherein both diploidization and impacts on female-specific differentiation are required (Ma et al. 2015; Verhulst et al. 2023). The two-step PI mechanism agrees with mounting evidence suggesting that a number of PI-*Wolbachia* strains manipulate the host's gene expression cascade that is responsible for female development (Verhulst et al. 2023). Furthermore, in related sexual lineages, female development relies on a paternally derived factor (Fig. 1A), the absence of which could pose a constraint for the asexual reproduction of females (Zou et al. 2020; Verhulst et al. 2023). Across Hymenoptera, this sexual differentiation pathway centers around the gene *transformer*, the primary signal for female development (Verhulst et al. 2010). The sex determination cascade involves alternative splicing of *transformer* transcripts to exclude a premature stop codon to produce a functional, female-specific Tra protein, TraF. Male-specific splicing yields a nonfunctional “TraM” protein resulting in male-specific splicing of downstream transcripts, and therefore male development. In the parasitoid wasp *Leptopilina clavipes*, wasps infected with *Wolbachia* strain “*w*Lcla” exhibit differential splicing compared to uninfected wasps, suggesting an interaction with the sex determination system, which could be the key to ensuring female development in such a two-step PI system (Geuverink et al. 2018).

Despite the discovery of microbe-mediated transitions to asexuality more than 30 yr ago (Stouthamer et al. 1990), the precise cellular mechanisms and bacterial genes responsible for manipulating host mitosis and reproductive biology have remained elusive. This is largely due to a paucity of genetic tools for *Wolbachia*, and, because PI-*Wolbachia* are not known to occur in genetically tractable models such as *Drosophila*, or even the model parasitoid *Nasonia.* Here, we focus on two-step PI-*Wolbachia*: *w*Lcla infecting *L. clavipes*, and *w*Tpre infecting *Trichogramma pretiosum* (Stouthamer and Kazmer 1994; Pannebakker et al. 2004). We hypothesized these *Wolbachia* would encode for an effector protein responsible for diploidization, and another responsible for driving female differentiation via interaction with the sex determination pathway. To test this hypothesis, we used comparative genomics across *Wolbachia* to identify two proteins, hereinafter termed PifA and PifB (for putative parthenogenesis-inducing factors A and B), and used molecular and functional assays to test their impacts on eukaryotic biology. Our results indicate that these are strong candidates as proteins responsible for the induction of parthenogenesis.

## Results

### Comparative Genomics Identifies Candidate Parthenogenesis-Inducing Factors (*pifs*)

We identified 10 sets of orthologous proteins (a total of 11 individual proteins from *w*Tpre and 20 from *w*Lcla) that were both (1) shared between *w*Lcla and *w*Tpre and (2) absent from all other *Wolbachia* strains in our dataset. Twenty-seven of these genes (eight complete orthogroups) were pseudogenized (supplementary table S6, Supplementary Material online). The remaining two sets of orthologous proteins were putatively functional, and each set contained one hypothetical protein from *w*Lcla and one from *w*Tpre. For simplicity, these two sets of 1:1 orthologs are hereinafter referred to as PifA and PifB, for putative parthenogenesis-inducing factors A and B.

While the clustering of orthologous *Wolbachia* proteins identified only *w*Tpre and *w*Lcla as having PifA and PifB orthologs, we considered that (A) these proteins might be found in the published PI-*Wolbachia* strains that we did not include in our database (e.g. the one-step PI strain *w*Uni; Gottlieb et al. 2002; Wang and Verhulst 2022), (B) there may be divergent Pifs present in other *Wolbachia* that did not meet clustering thresholds, (C) there might be unannotated *pif*-like regions present in other *Wolbachia* genomes, and (D) there may be Pif-like proteins in other organisms. To address some of these issues, we used blast to search for putative Pif homologs across non-redundant NCBI databases. Both protein and nucleic acid searches (blastp, tblastn, blastn, and discontiguous megablast) of *pifA* homologs only recovered matches to the already identified *w*Tpre and *w*Lcla PifA loci indicating that these are unique genes restricted to these PI-*Wolbachia* strains. Protein blast (blastp) searches of PifB identified short regions of distant homology to other proteins that contained leucine-rich repeat (LRR)-like domains. Outside of the *w*Tpre and *w*Lcla PifB orthologs, the highest scoring hit was a protein from an Alphaproteobacterium that aligned to 19% of the PifB sequence (at the LRR domains), with 30% amino acid identity (supplementary fig. S1 and table S11, Supplementary Material online). Lower scoring hits included a small number of proteins from other *Wolbachia* that had also acquired LRR-like domains, but, like the other database matches, they did not appear to be homologs of the *w*Tpre and *w*Lcla PifB proteins. Regions of similarity were divergent and restricted to the LRR-like domains, and the proteins did not globally align to the *w*Tpre and *w*Lcla Pifs or have any nucleotide-level sequence homology (supplementary fig. S1 and table S11, Supplementary Material online).

Both PifA and PifB have canonical features of eukaryotic host-associated bacterial effector proteins, including the aforementioned restricted phylogenetic patterns. Additionally, there are several shared characteristics with *Wolbachia* proteins that mediate other reproductive phenotypes. These similarities include proximity to phage and other mobile element loci, and location within a putative eukaryotic association module (EAM; Fig. 1C). The EAM is a region found in many *Wolbachia* genomes: it is associated with *Wolbachia* prophage (WO) regions or remnants, and in other *Wolbachia* strains the EAM contains loci responsible for cytoplasmic incompatibility and/or male-killing (Bordenstein and Bordenstein 2016; LePage et al. 2017; Lindsey et al. 2018; Perlmutter et al. 2019). *Trichogramma*-infecting *Wolbachia* were previously identified as having lost their WO phages (Gavotte et al. 2007), and genome sequencing of the *w*Tpre strain revealed only degenerate prophage WO regions (Lindsey et al 2016). Indeed, the *pif* loci in *w*Tpre are surrounded by those degenerate phage and transposable element loci, as well as other pseudogenes consistently found in the EAM, including *mutL*, a patatin-like gene, transcriptional regulators, and a suite of ankyrin domain containing proteins (Fig. 1C). The homologous *pif* loci in *w*Lcla are contained within a short contig (wLcla_Contig_2; ∼11 kb) that also includes transposases, a transcriptional regulator, and pseudogenized hypothetical proteins. Due to the fragmented nature of the *w*Lcla assembly, it is difficult to determine if the *w*Lcla *pif* homologs are in a true prophage and/or EAM-like region. Finally, while *pifA* and *pifB* are syntenic in *w*Tpre (329 bp between the 3′ end of *pifA* and the 5′ end of *pifB*), in *w*Lcla the two genes are in opposite orientations and separated by a ∼1.6 kb region that contains a pseudogenized transposase and two small hypothetical proteins.

**Fig. 2.**
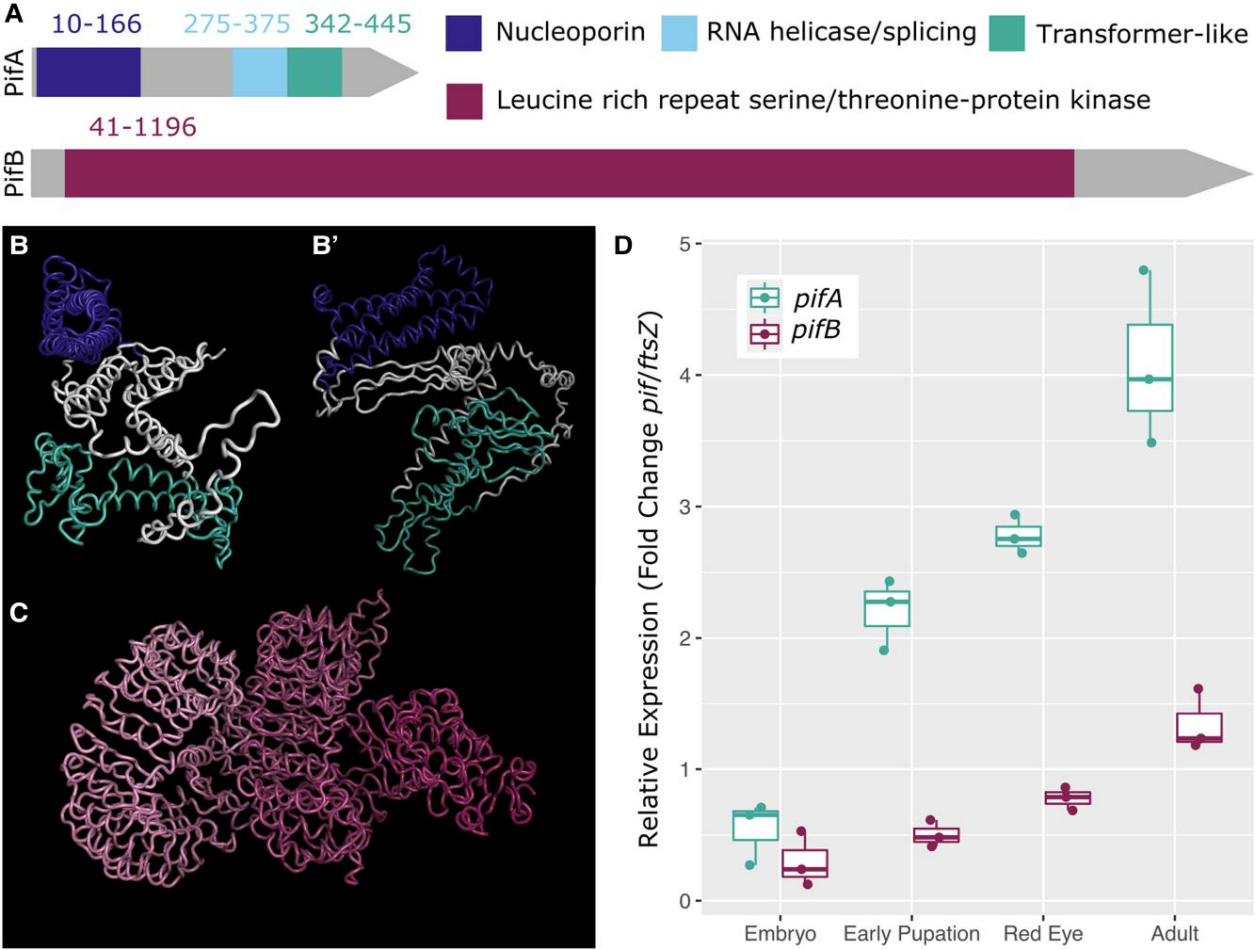
Candidate parthenogenesis-inducing factors PifA and PifB. A) Domain prediction indicates PifA and PifB contain eukaryotic-like structures. Colored coordinates indicate amino acid positions of the relevant domain in the *w*Tpre ortholog. B and B′) Two views of the predicted structure of the PifA protein from *w*Tpre. B″ view is rotated 90° clockwise around the vertical axis as compared to the view in (B). C) Predicted structure of the PifB protein from *w*Tpre. D) *pif* expression (relative to a bacterial housekeeping gene) increases across wasp development in *T. pretiosum*.

PifA and PifB contain eukaryotic-like domain structures: another key indicator of bacterial proteins that manipulate eukaryotic biology. Structural prediction revealed four regions of the Pif proteins with significant structural homology to eukaryotic domains (Fig. 2A; supplementary tables S7–S10, Supplementary Material online). The first is a domain with high similarity to nucleoporins at the N-terminus of PifA. Additionally, PifA contains two overlapping domains predicted to function in RNA splicing. Most notably, the second of these has significant structural similarity (33%) to the insect protein Transformer (Tra), discussed above for its role as the master-regulatory sex determination gene (Verhulst et al. 2010). Finally, PifB is primarily composed of a large LRR domain with strong structural homology to eukaryotic LRR receptor-like serine/threonine protein kinases, especially membrane-bound Toll-like receptors (Fig. 2A). *w*Tpre homologs of the Pif proteins were modeled with Robetta to visualize putative 3D protein structures (Fig. 2B and C; supplementary files S1–S4, Supplementary Material online). The predicted PifA protein structure consisted of a pore-like formation in the nucleoporin-like region (error estimate <10) and a relatively unstructured region aligning with the predicted RNA splicing/Tra-like domain. PifB structure results produced a double coiled structure typical of LRRs. Lastly, PifA and PifB are predicted to be bacterial secretion substrates, with a strong likelihood of secretion via the type IV secretion system, which is known to translocate *Wolbachia* effector proteins to the host in other strains (Whitaker et al. 2016) (supplementary table S12, Supplementary Material online).

### *Wolbachia* Infection Is Correlated With Differential Splicing of *Transformer* and *Double-Sex*

Given the discovery of a Tra-like *Wolbachia* protein, and data indicating that *Wolbachia* strain *w*Lcla impacts sex-specific splicing of *tra* in *L. clavipes* females (Geuverink et al. 2018), we queried previously published RNA-seq data from adult female *T. pretiosum* to assess the impacts of *Wolbachia* on *tra* and *dsx* splicing. These data are derived from three sets of wasp colonies, each with a genetically identical, *Wolbachia*-infected and -uninfected pair (*n* = 6 total). Exon use was previously assessed genome-wide, and here we display these patterns specifically for *tra* and *dsx* (Fig. 1D and E). We find that in female wasps without *Wolbachia*, exon 5 of *tra* and exons 3 and 5 of *dsx* are uniquely excluded. However, while female wasps with *Wolbachia* do use *tra* exon 5, there was no identifiable expression of *tra* exons 1 and 2.

### *pif* Expression Correlates With Insect Reproduction

To see if *pifA* and *pifB* are expressed, and thus less likely to be an artifact of genome annotation, we used reverse transcription PCR (RT-PCR) to amplify *pifA* and *pifB* transcripts from a pool of ∼2–4-h old *T. pretiosum* embryos, and a pool of adult females. Indeed, both *pifA* and *pifB* are expressed in adult females, and we detected low or no expression of *pifA* and *pifB* in the young embryos (supplementary fig. S2, Supplementary Material online). Because *pifA* and *pifB* are syntenic in *w*Tpre, reminiscent of the cytoplasmic incompatibility loci which are co-transcribed (Lindsey et al. 2018), we also used RT-PCR to look for the presence of *pifA–pifB* monocistronic transcripts, indicative of an operon. We were not able to amplify any monocistronic transcripts from adult females using primers that targeted the 5′ region of *pifA* and the 3′ region of *pifB* (supplementary fig. S3, Supplementary Material online).

To determine if *pif* expression correlated with reproductive biology, we used quantitative reverse transcription PCR (qRT-PCR) to quantify relative *pifA* and *pifB* expression across four time points in *T. pretiosum* development: early in embryogenesis, during the transition to metamorphosis, the “red-eye stage” of metamorphosis during which gonads undergo differentiation, and in adult females (Fig. 2D). There was a statistically significant increase in both *pifA* and *pifB* expression across wasp development (*F*_1,3_ = 61.4, *P* < 0.0001). However, the relative expression of *pifA* was always significantly higher than *pifB* post-embryonic development (*F*_1,3_ = 18.51, *P* < 0.0001). At 2–4 h post-parasitization (shortly after the first mitotic division during which the *Wolbachia*-mediated gamete duplication step occurs; Stouthamer and Kazmer 1994; Pannebakker et al. 2004), there was minimal expression of *pif* genes. However, and importantly, these are measurements of mRNAs, so there may well be maternally loaded Pif proteins in these embryos. In freshly emerged adult females, expression of *pifA* and *pifB* was 4- and 1.5-fold higher than in embryos, respectively. Expression patterns of *pifA* and *pifB* in *w*Lcla and *L. clavipes* recapitulated these patterns, though *pifA* had even greater expression than *pifB* (up to 25× higher; supplementary fig. S4, Supplementary Material online).

### PifA Is Conserved and Repetitive in the Tra-Like Region

**Fig. 3.**
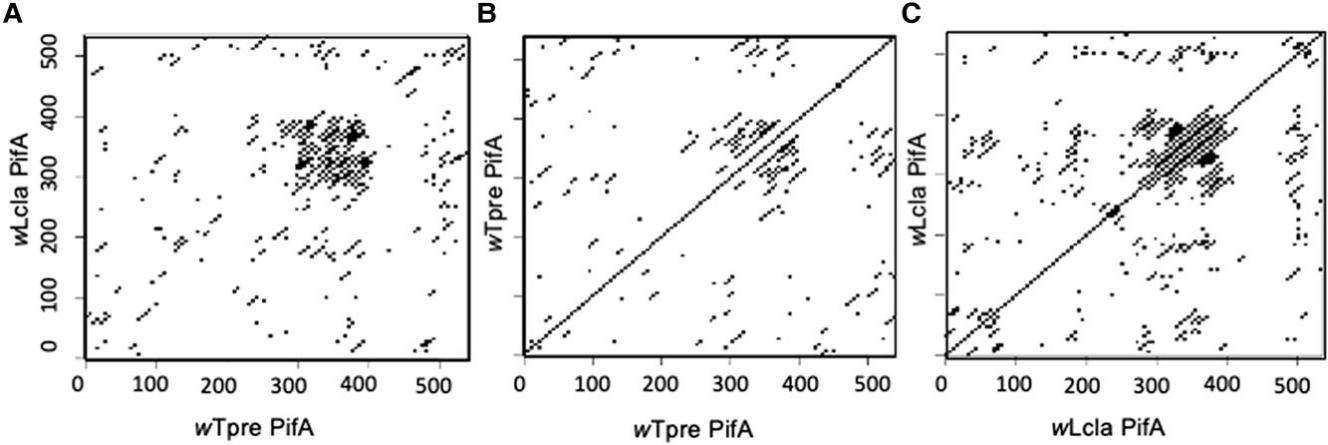
PifA is conserved and repetitive in the Tra-like region. Dotplots showing amino acid similarity, where each point represents a window of five identical amino acids. A) *w*Tpre PifA versus *w*Lcla PifA, showing high conservation between the orthologs in the ∼320–420 amino acid region. B) *w*Tpre PifA versus self, showing a series of repeated regions in the Tra-like region. C) *w*Lcla PifA versus self, showing a series of repeated regions in the Tra-like region.

In validating plasmids and sequencing entry amplicons (see Methods), we discovered that the *w*Tpre *pifA* gene (supplementary file S5, Supplementary Material online) contains a short region present in tandem triplicate that had been collapsed into a duplicate during genome assembly. The *w*Lcla PifA ortholog was assembled correctly, based on our amplicon sequencing, though there were similar repeated regions in the same part of the coding sequence. Given these findings on the repetitive nature of PifA, we next characterized these patterns and their conservation. First, we compared the two orthologs to each other and we found a region of high similarity aligning with the Tra-like domain (Fig. 3A). Here, there is a ∼100 amino acid region of high synteny and conservation. This conserved region itself composed of tandemly repeated blocks of 12–18 amino acids (seen in the classic clustering of the dot plot in that region; Fig. 3A). To better visualize these tandem repeats, we compared each ortholog to itself (Fig. 3B and C). In *w*Tpre, there are 4 of repeated segments in total, each 18 amino acids long (Fig. 3B). In *w*Lcla, the repeated region was composed of 6 segments, each 12 amino acids long, and there was an additional instance of that repeated segment in the ∼250 amino acid region (Fig. 3C).

### PifA and PifB Impact Eukaryotic Cells

**Fig. 4.**
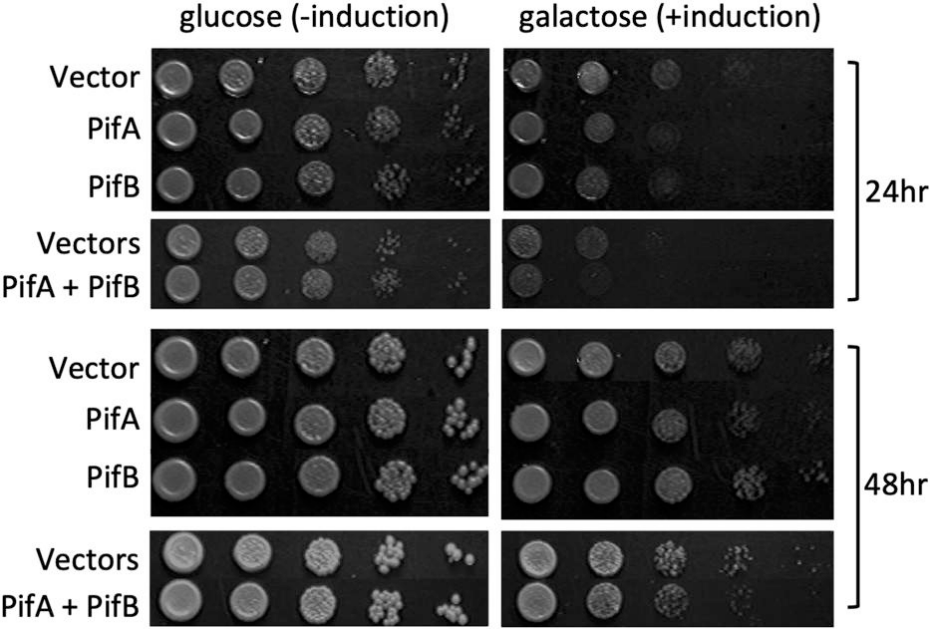
PifA and PifB impact eukaryotic cell biology. PifA and PifB cause growth defects in yeast relative to empty vector and no induction controls. Yeast strain W303 MATa was transformed with galactose-inducible 2-μm expression vectors that were either empty or contained a Pif coding sequence. Transformants were grown up in selective non-inducing media, normalized to OD600 = 1, and serial 1:10 dilutions were spotted on selective inducing or non-inducing plates and grown at 30 °C for 48 h. Single vector conditions leveraged the pAG425 backbone, and double expression conditions leveraged both pAG424 and pAG425 (see Supplementary material online).

To determine if PifA and PifB are putative effector proteins, we used a standard screen in the budding yeast, *Saccharomyces cerevisiae*. Bacterial effector proteins that interact with eukaryotic cell biology often cause growth defects when expressed in yeast cells (Kramer et al. 2007; Siggers and Lesser 2008; Sheehan et al. 2016; Beckmann et al. 2017; Rice et al. 2017). We expressed the *w*Tpre PifA and PifB genes in W303 MATa yeast using a 2-μm expression vector with a galactose-inducible expression system, pAG424GAL-ccdB and/or pAG425GAL-ccdB (Alberti et al. 2007; Fig. 4). We additionally co-expressed PifA and PifB to test if there were synergistic or interactive effects on the yeast, which might indicate physical interaction between the two proteins, or impacts on the same structures or pathway. At 24 h, there were identifiable growth defects in the yeast expressing PifA or PifB, relative to the empty vector control. Co-expression of both Pifs also caused growth defects relative to the double vector control. We note that the yeast transformed with two high copy number 2-μm expression vectors generally do not grow as well, but there are still clear differences between the empty and Pif-containing conditions. By 48 h, there was still evidence for negative impacts on yeast growth in the PifA and co-expressing yeast. However, the yeast expressing only PifB were more similar to the empty vector controls at this later timepoint.

### PifA Localizes With Atypically Dividing Nuclei

**Fig. 5.**
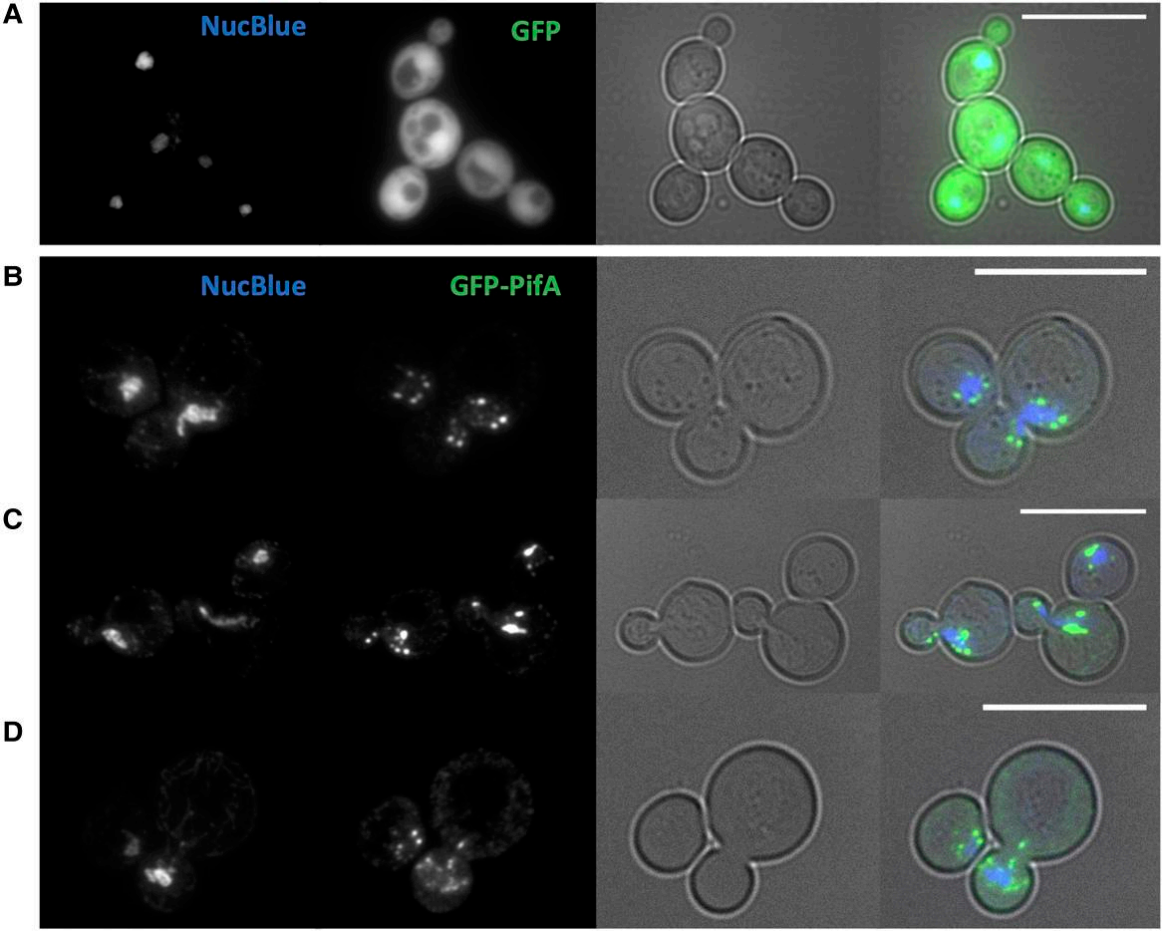
PifA associates with dividing nuclei. To localize PifA proteins, yeast W303 MATa were transformed with galactose-inducible pFUS expression vectors (with an N-terminal GFP tag) and grown in selective inducing media (with 2% galactose as a carbon source) for 6 h, and stained with NucBlue. Scale bars represent 10 μm. A) Yeast with empty pFus, expressing GFP alone. B–D) Yeast with pFus-PifA, expressing GFP-PifA.

Finally, given the stronger growth impacts of PifA, and its nucleoporin-like domain, we used a fluorescent fusion tag (Green Fluorescent Protein; GFP) to localize PifA within the yeast cells. GFP alone displayed a homogeneous localization pattern spread evenly throughout the yeast cytoplasm (Fig. 5A). In contrast, GFP-PifA exhibited strong nuclear or perinuclear localization, reminiscent of localization patterns of known nucleolar proteins (Carmo-Fonseca et al. 2000; Taddei and Gasser 2012; Fig. 5B–D). Some yeast cells exhibited GFP-PifA puncti near each pole of dividing nuclei and deposition into the daughter cell (Fig. 5B and C). Other yeast cells had what looked like PifA dispersed around the dividing nucleus (Fig. 5D). Importantly, there was also evidence for atypical mitoses in the presence of PifA (Fig. 5D) with uneven distribution of daughter nuclei in the two cells.

## Discussion

For decades the genes responsible for PI have remained elusive. This is despite increasing examples of microbe-mediated PI in nature, and significant progress made in understanding the mechanisms of other microbial reproductive manipulations such as cytoplasmic incompatibility and male-killing (Kaur et al. 2021). Indeed, PI is present in *Wolbachia* strains infecting at least six separate genera of parasitoid wasps (Ma and Schwander 2017). Because PI evolved multiple times across *Wolbachia*, we focused on strains predicted to share the same two-step mechanism of PI. As such, we identified and characterized two *Wolbachia* proteins that we predict are involved in the induction of parthenogenesis in *T. pretiosum* and *L. clavipes:* PifA and PifB.

Broadly, the proteins we identified have many characteristics of host-associated bacterial effector proteins. The *pif* genes have expression patterns that are coordinated with wasp development, with little to no expression in embryos, and high expression in adult females. We hypothesize there are maternally loaded Pifs present in embryos, and limited additional transcription happening, which correlates with the low levels of embryonic *pif* mRNA. Additionally, the short time window in which diploidization occurs likely necessitates pre-loading of the embryos with the appropriate *Wolbachia* effectors. Further support for the role of PifA and PifB as effector proteins includes growth phenotypes in yeast, and predictions that the Pifs are type IV secretion substrates (bacterial protein translocation systems that both *w*Lcla and *w*Tpre encode for; Lindsey 2020). We do note that yeast and insect mitoses have a number of key differences (e.g. open vs. closed; Sazer et al. 2014), and any signal indicating egg fertilization would likely not manifest in yeast cells, so it is unsurprising that the growth impacts in yeast are more mild than many other pathogenic bacterial effector proteins with broad host ranges (e.g. in *Legionella*; Siggers and Lesser 2008).

Regardless, the predicted domain structures of the Pif proteins are highly characteristic of functions in eukaryotic cells. PifA has both a nucleoporin-like region and an RNA-splicing-like region with homology to the insect-specific protein Transformer which is a strong indicator of interaction with host sex determination. The structural homologies of PifB to serine/threonine kinases, LRRs, F-box, and Toll-like receptor motifs indicate the ability to recognize, bind, and/or alter other proteins. LRR motifs are widespread in eukaryotic proteins and are typically involved in mediating protein–protein interactions (Kobe and Kajava 2001). Consequently, LRR motifs are also found in other bacterial effector proteins, acting to mimic and interface with host biology (Aepfelbacher et al. 2005; Xu et al. 2008).

In line with our hypotheses about the diversity of mechanisms underlying PI, there is no evidence for these proteins in other published *Wolbachia* genomes, including strains that use other PI mechanisms (e.g. the one-step PI strain *w*Uni; Wang and Verhulst 2022), based on blast results. Results from computational predictions for PifA and PifB also align with our expectation that two-step PI-*Wolbachia* need machinery for interacting with both chromosome division and the sex determination system. In two-step PI systems, the diploidization event during early embryogenesis is critical. This diploidization (in *w*Tpre and *w*Lcla, the failed anaphase) appears to be highly regulated as it only ever occurs during the first embryonic mitosis. If diploidization does not occur at the first mitosis (perhaps due to reduced *Wolbachia* titers), then it does not appear to happen at all (Tulgetske 2010). Additionally, PI-*Wolbachia* do not appear to cause rampant polyploidy during development, and *Wolbachia*-mediated diploidization does not occur in infected embryos if they have been fertilized (Stouthamer and Kazmer 1994). In many *Trichogramma*, PI-*Wolbachia*-infected females can mate and fertilize their eggs, in which case, the resulting offspring are diploid females and not triploid offspring (Stouthamer and Kazmer 1994), indicating that the PI mechanism is sensitive to both the specific mitotic event and to whether the egg has been fertilized.

Considering the specific conditions in which diploidization takes place, we hypothesize there is a host factor that serves as a signal for both the timing of the diploidization event (the first mitosis) and if diploidization is “not needed” (i.e. because the egg was fertilized). We argue it is unlikely for embryo ploidy itself to directly serve as this signal, for several reasons. First, in insects, maternal and paternal pronuclei stay physically separated for most of the first mitotic cycle (Tram et al. 2003). Second, even if *Wolbachia* or PI factors were capable of “counting chromosomes”, a ploidy signal would not explain why the diploidization event only ever occurs at the first mitosis. Finally, interactions with a separate male sex-ratio distorter that is present in some *Trichogramma* species, the “paternal sex-ratio” (PSR) chromosome, indicate *Wolbachia*-mediated PI is sensitive to fertilization independent of ploidy. Specifically, the PSR “B-chromosome” is transmitted by haploid males: upon fertilization, the PSR chromosome mediates destruction of all other paternal chromosomes, resulting in a haploid male (a single maternal genome copy) plus the PSR chromosome which can then be transmitted to the next generation. Critically, when these PSR-males mate with *Wolbachia*-infected females, PSR-mediated distortions take precedence over *Wolbachia*-mediated PI (Stouthamer et al. 2001). The paternal chromosomes are destroyed, leaving a haploid embryo with PSR, but *Wolbachia-*mediated diploidization never occurs. Thus, there is likely a non-chromosomal factor associated with fertilization, or its absence, that ultimately makes *Wolbachia*-mediated PI in this system possible both (1) during a specific mitotic event and (2) in the absence of fertilization.

A potential non-chromosomal factor that could signal both conditions is the presence or absence of paternally contributed centrioles. In fertilized hymenopteran embryos (e.g. *Nasonia vitripennis*), the sperm-tail basal body preferentially nucleates paternal centrioles to take part in the first mitotic division, and maternal cytoplasmic asters disperse (Ferree et al. 2008). In unfertilized, haploid embryos, this process is compensated for by de novo centrosome formation in the embryo from the maternal cytoplasmic asters (Ferree et al. 2006). The absence of a paternal centrosome in unfertilized eggs may serve as a signal or is possibly functionally significant in the PI mechanism. In the unfertilized embryo, if the PI proteins interact with the de novo formation of centrosomes, inhibition of proper chromosome segregation may result. Indeed, the centrosome looks to be a target of other sex-ratio modifying infections such as the male-killing bacterium *Arsenophonus nasoniae* in *N. vitripennis*. Here, the bacterium suppresses maternal centrosome formation in the unfertilized, male destined embryos. This results in mitotic spindle disruption, failed development of the early embryo, and ultimately male-specific mortality (Ferree et al. 2008). In this system, a PSR chromosome can also rescue the *Arsenophonus* male-killing phenotype, again pointing to potentially similar targets (Ferree et al. 2008).

Regardless of what the specific target of PI-*Wolbachia*-mediated diploidization may be, we do know that an extensive network of protein kinases is required for successful mitosis in animal cells. These protein kinases, such as Aurora A, Aurora B, and polo kinase in *Drosophila melanogaster*, have multiple roles in regulating spindle assembly, centrosomal function, and cytokinesis (Archambault and Carmena 2012; Reinhardt and Yaffe 2013). The serine/threonine protein kinase-like domain of PifB may indicate participation in these mitotic processes. Additionally, in many eukaryotes, mitotic phosphorylation sites typically lie in serine/threonine motifs (Canova and Molle 2014). Alternatively, PifA may be participating in mitotic disruption, as the N-terminal region has structural homology to a suite of nucleoporins that drive spindle assembly and chromosome organization in a wide range of other organisms (Chatel and Fahrenkrog 2011). Indeed, many of the PifA localization patterns in dividing yeast cells (Fig. 5) are reminiscent of the nucleoporin ALADIN in *D. melanogaster* (Carvalhal et al. 2015). ALADIN aggregates at the poles of dividing nuclei, and regulates localization of Aurora A (a serine/threonine kinase) and spindle organization (Carvalhal et al. 2015). There is also potential for two Pif proteins to interact to drive the failed first anaphase. For example, one protein's activity might be sensitive to the presence of the critical host factor (e.g. perhaps centrioles, as discussed above), and the other protein could then recruit the first protein to the critical host structures. This is of course speculation, but the localization patterns of PifA and mitotic impacts hint at more than interaction with the sex determination pathway.

In addition to this tightly regulated diploidization event, a second step is required to achieve female development in the *w*Lcla and *w*Tpre PI systems. In many sexual hymenopterans, female development seems to rely on a paternally derived signal. This might be in combination with a maternal effect (as in *N. vitripennis*; Beukeboom and Van De Zande 2010), or the paternal signal might be sufficient on its own (as is predicted to be the case in *L. clavipes*; Chen 2021). The absence of a paternal signal during asexual reproduction thus presents a constraint on female development. In the sexual species, *N. vitripennis*, the maternal *tra* allele is transcriptionally repressed in the embryo via an upstream factor, *wom*, and a combination of maternally loaded *traF* mRNAs and a transcriptionally active paternal *tra* allele is necessary for female development (Beukeboom and Van De Zande 2010; Zou et al. 2020). In contrast, maternally provided *traF* is absent in *Wolbachia-*uninfected *L. clavipes* embryos (Chen 2021), and it may be the case that paternal activation of the pathway or upstream factor that leads to *traF* is sufficient. Importantly, *tra* isoforms differ between *Wolbachia*-infected and *Wolbachia*-uninfected adult female *L. clavipes*. In the *Wolbachia*-infected wasps, only the *traF* variant is present, as compared to uninfected wasps where both *traF* and *traM* splice variants are present (Geuverink et al. 2018). Our data similarly indicate that *Wolbachia* is acting on the maternal pool of *tra* and *dsx* isoforms (Fig. 1D and E); this perhaps could be a result of the high expression of PifA at that timepoint in adult females (Fig. 2D).

The Tra-like domain within PifA may function as a replacement to initiate *tra* autoregulation and/or act on downstream sex determination genes such as *dsx* to ensure female differentiation. The nuclear localization of PifA in yeast cells notably complements its predicted nucleoporin-like region, suggesting that it may function within the host nucleus or perhaps on the nuclear membrane where post-transcriptional processing often occurs (Keene 2007). Indeed, previous experiments in *T. pretiosum* found that *Wolbachia* infection results in a number of differentially expressed and differentially spliced transcripts, and included in these are genes in the sex determination pathway (Wu et al. 2020; Fig. 1D and E). Given the predicted structure of PifA, these patterns could be attributed to direct splicing of host RNAs. However, it is also possible that PifA may act via allosteric or competitive interactions with the spliceosome, other RNA binding proteins, or other upstream processes.

PI microbes are clearly rich in factors for manipulating chromosome biology and sex determination of their hosts. Considering the impact of PifA and PifB on yeast biology, their location in a EAM genomic region, and structural similarities to eukaryotic domains involved in mitosis and sex determination, these proteins are promising candidates for PI. At present, we are quite limited by the lack of genetic tools in *Wolbachia* and natural hosts of PI-*Wolbachia*. Investigation of other *Wolbachia* phenotypes such as cytoplasmic incompatibility and male-killing has leveraged accessible heterologous expression systems in the *D. melanogaster* model: an insect that naturally harbors such symbionts. Indeed, tools in parasitic hymenopterans or other haplodiploid arthropods are quite sparse. Even in the most well-studied species, *N. vitripennis*, clustered regularly interspaced short palindromic repeats has only recently been used for gene-editing (Li et al. 2017; Chaverra-Rodriguez et al. 2020), but as of yet no one has achieved integration of a transgene in any Hymenopteran. Ongoing investigation into the mechanisms of symbiont-mediated PI and deciphering the involvement of PifA and PifB will thus require a stronger focus on biochemical approaches and methods development for cell biology and genetics in these difficult to work with insects. Regardless, our study represents the first identification of putative parthenogenesis-inducing proteins, a major step toward understanding host-symbiont interactions and the evolution of asexual reproduction.

## Materials and Methods

### Genomics, Domain Prediction, and Protein Modeling

Thirty-three complete or near-complete *Wolbachia* genome assemblies representing nine supergroups (A, B, C, D, E, F, J, L, T) were used in comparative genomic analyses to identify putative parthenogenesis-inducing factors (supplementary table S1, Supplementary Material online). We specifically included the two PI-*Wolbachia* strains with a shared cytological mechanism for PI, *w*Tpre and *w*Lcla that infect the parasitoid wasps *T. pretiosum* and *L. clavipes*, respectively (Stouthamer and Kazmer 1994; Pannebakker et al. 2004). All genome sequences and prokaryotic genome annotation pipeline “RefSeq” annotations (Tatusova et al. 2016) were used to build orthologous groups of *Wolbachia* proteins using ProteinOrtho v5.15 with default parameters (Lechner et al. 2011). We identified groups of orthologous genes that were unique to the two PI-*Wolbachia*. Protein domain prediction was performed with HHpred (Söding et al. 2005; Zimmermann et al. 2018) using SMART_v6.0, PDB_mmCIF70_12_Oct, and Pfam-A_v34 as reference databases. Significant matches to structural domains were determined as those with probabilities greater than 50% or greater than 30% and in the top five hits, as per published HHpred best practices (Söding et al. 2005; Zimmermann et al. 2018). 3D protein modeling was performed with Robetta's RoseTTAFold (Baek et al. 2021). Structures were visualized in PyMol (The PyMOL Molecular Graphics System, Version 2.0 Schrödinger, LLC). Secretion substrate prediction was performed with BastionX (Wang et al. 2021) and EffectiveDB (Eichinger et al. 2016). Dotplots were created in RStudio using the seqinr package, with parameters wsize = 20, wstep = 5, and nmatch = 5. Previously published RNA-seq data (Wu et al. 2020) were queried to visualize exon usage of *transformer* and *double-sex* transcripts in genetically matched female *T. pretiosum* with and without their *Wolbachia* infections (asexual and sexual, respectively). Supplementary table S2, Supplementary Material online, contains information on the annotations and accession numbers for the sex determining loci.

### Insect Rearing and Developmental Series

All insects were maintained at 25 °C on a 24-h, 12:12 light:dark cycle. Wasp colonies included: *T. pretiosum* “Insectary line” infected with *Wolbachia* strain *w*Tpre (Lindsey et al. 2016, 2018) and *L. clavipes* “LcNet” infected with *Wolbachia* strain *w*Lcla (Pannebakker et al. 2004; Kraaijeveld et al. 2016; Kampfraath et al. 2019; Lue et al. 2021). *L. clavipes* were hosted on *Drosophila virilis* (National Drosophila Species Stock Center SKU: 15010-1051.87), maintained on standard Bloomington cornmeal-agar medium (Nutri-fly Bloomington Formulation) under density-controlled conditions. *T. pretiosum* were hosted on UV irradiated eggs of *Ephestia kuehniella* (Beneficial Insectary).

To compare the expression of Pifs across host development, we generated a synchronized developmental series for each wasp species. Adult *T. pretiosum* were given egg cards (*E. kuehniella* eggs adhered to cardstock with double-sided tape) for 2 h. Adult wasps were removed, and egg cards were held under standard rearing conditions until collection. Time points included: (1) 2–4 h post-parasitism, during the embryo stage, (2) three days post-parasitism, at the prepupal stage, when host *Ephestia* eggs turned gray, (3) seven days post-parasitism, at the red-eye pupal stage when ovaries are maturing, and (4) adult females less than 24 h post-eclosion that had not accessed fresh host eggs. Sampling times were based on published developmental studies of *Trichogramma* (Flanders 1937; Volkoff and Daumal 1994; Volkoff et al. 1995; Jarjees and Merritt 2002). Samples were collected in biological triplicate, and flash frozen by placement at −80 °C prior to further processing (see below). To prepare a synchronized developmental series of *L. clavipes*, one-day-old adult female wasps were given early second-instar *D. virilis* larvae and observed for parasitism. Parasitized fly larvae were left to develop either for (1) 2 h (embryo), (2) 14 days (early wasp pupation), or (3) 18 days (red-eye wasp pupae). Adult females were collected for gene expression analyses within 24 h of eclosion and prior to host access. Each stage was collected in biological triplicate as pools of three individuals, except for the embryo stage in which pools were composed of six individuals, to adjust for the small size. Samples were flash frozen and stored at −80 °C for further processing.

### Nucleotide Extractions, PCR, RT-PCR, and qRT-PCR

DNA was extracted from flash-frozen samples with the Monarch Genomic DNA Purification Kit (New England Biolabs), including the on-column RNase treatment. RNA was extracted from flash-frozen samples with the Monarch Total RNA Miniprep Kit (New England Biolabs) including the DNase treatment step. The LunaScript RT Master Mix Kit was used for making cDNA, following manufacturer's instructions. PCR reactions were performed with Q5 Hot Start High-Fidelity 2× Master Mix (New England Biolabs) in 20 µl reactions and products were run on a 1% agarose gel alongside an appropriately sized ladder (either New England Biolabs 100 bp or 1 kb DNA ladder) and stained post-electrophoresis with GelRed (Biotium). *pif* expression was assessed with the Luna Universal Two-Step RT-qPCR Master Mix (New England Biolabs) following manufacturer's instructions, and normalization to *ftsZ* (*Wolbachia* housekeeping gene). All reactions were run in technical duplicate alongside a standard curve and negative controls (including no template and no reverse transcriptase controls) on a QuantStudio 3 Real-Time PCR System (Applied Biosystems). All primer sequences are in supplementary table S3, Supplementary Material online.

### Cloning and Yeast Transformation

To assess the impacts of Pifs on eukaryotic cells, we expressed them in the budding yeast *S. cerevisiae*. Coding sequences of *pifA* and *pifB* were PCR amplified from *w*Tpre-infected wasps using modified forward primers to facilitate cloning into pENTR-D/TOPO (Invitrogen). These constructs were transformed into One Shot Top10 competent cells (Invitrogen) following manufacturer's instructions (see supplementary table S3, Supplementary Material online for primer sequences) and plated on selective media. Entry vectors were verified first with restriction digests, followed by whole-plasmid sequencing (Plasmidsaurus). Validated entry vectors were recombined into an appropriate destination vector (see supplementary table S4, Supplementary Material online) in an LR clonase reaction following manufacturer's instructions (Invitrogen). Expression vectors were cloned into One Shot Top10 competent cells (Invitrogen), plated on selective media, and validated as above. Yeast strain W303 MATa (supplementary table S5, Supplementary Material online) was transformed with expression vector(s) using the polyethylene glycol/Lithium acetate method (Yeast Transformation Kit, MilliporeSigma; Gietz and Woods 2002) and plated on non-inducing selective synthetic media (Sigma-Aldrich Yeast Synthetic Drop-Out Media Supplements). In all cases, plasmid extractions from *Escherichia coli* were performed with the Monarch Plasmid Miniprep Kit (New England Biolabs). Yeast transformants were grown overnight at 30 °C in selective non-inducing media with 2% glucose as a carbon source. Cells were harvested by centrifugation and washed twice with sterile water, followed by resuspension in water to an OD_600_ of 1.0. A five step, ten-fold dilution series was prepared for each transformant and 3 µl spots were plated on either selective inducing (2% galactose) or selective repressing (2% glucose) media. After spot plates were dried, they were incubated at 30 °C for 48 h.

### Microscopy and Image Analysis

For fluorescence imaging, yeast transformed with GFP-tagged Pifs (or GFP alone) were grown overnight at 30 °C in selective non-inducing media, supplemented with 2% raffinose as a carbon source, and 20µg/ml of additional adenine to minimize autofluorescence (Rines et al. 2011). Cells were harvested via centrifugation and resuspended in selective inducing media (2% galactose) prior to incubation at 30 °C for an additional 6 h. After the 6-h induction period, yeast cells were stained with NucBlue Live ReadyProbes Reagent (Hoechst 33342; Invitrogen). Images were taken on an ECHO Revolve Fluorescence Microscope fitted with an 100 × Apochromat Oil Immersion lens, fluorescein isothiocyanate and 4',6-diamidino-2-phenylindole light emitting diode filter sets, and an extra long working distance Universal Condenser. Images were merged and pseudocolored in FIJI (Schindelin et al. 2012).

### Statistics and Data Visualization

Statistical analysis and data visualization were performed in R 4.1.3 (R Core Team 2014). Variation in *pif* expression was assessed with a two-way analysis of variance including gene and developmental stage as fixed effects, followed by post hoc testing with the Tukey–Kramer method. Schematics were created with BioRender.com and data visualization leveraged the “muted qualitative colour scheme” designed by Paul Tol (https://personal.sron.nl/∼pault/).

## Supplementary Material

evae036_Supplementary_Data

## Data Availability

The data underlying this article are available in the article and in its Supplementary material online.
