# Supplementary material for: Identification of Parthenogenesis-Inducing Effector Proteins in Wolbachia
Source: Genome Biol Evol. 2024 Mar 26;16(4):evae036. doi: 10.1093/gbe/evae036 (PMC11019157; doi:10.1093/gbe/evae036)
Supplement: evae036_Supplementary_Data [file evae036_supplementary_data.zip › List of Supplemental Materials.docx]

Supplemental Excel File Contains:

Table S1. Genomes used in comparative analyses

Table S2. Sex determining genes in *Trichogramma pretiosum*

Table S3. Primers

Table S4. Vectors

Table S5. Yeast strains

Table S6. Orthogroups unique to *w*Tpre and *w*Lcla

Table S7. HHpred results for *w*Tpre PifA

Table S8. HHpred results for *w*Tpre PifB

Table S9. HHpred results for *w*Lcla PifA

Table S10. HHpred results for *w*Lcla PifB

Table S11. PifB BLASTP Results

Table S12. Secretion prediction results for wLcla and wTpre PifA and PifB homologs

Supplemental File S1 Contains:

Figure S1. PifB BLASTP matches outside of *w*Tpre and *w*Lcla are restricted to low similarity LRR-like domains in other bacteria.

Figure S2. *pifA* and *pifB* are expressed in adult female *Trichogramma pretiosum*.

Figure S3. *pifA* and *pifB* are not co-transcribed in *Trichogramma pretiosum.*

Figure S4. Time series of *w*Lcla *pifA* and *pifB* expression in *Leptopilina clavipes*.

Other Supplemental Files:

Supplemental File S2: Robetta results for PifA

Supplemental File S3: Robetta results for PifB with 29 N-terminal amino acids deleted

Supplemental File S4: Robetta results for PifA with 29 C-terminal amino acids deleted

Supplemental File S5: Fasta file containing the corrected wTpre PifA sequence
