# Supplementary material for: Identification of Parthenogenesis-Inducing Effector Proteins in Wolbachia
Source: Genome Biol Evol. 2024 Mar 26;16(4):evae036. doi: 10.1093/gbe/evae036 (PMC11019157; doi:10.1093/gbe/evae036)
Supplement: evae036_Supplementary_Data [file evae036_supplementary_data.zip › pifs_FileS1_supplFigs_20240206.pdf]

Supplemental Figures For:
Identifying parthenogenesis-inducing effector proteins in *Wolbachia*

Laura C Fricke[1] and Amelia RI Lindsey[1*]
[*]To whom correspondence should be addressed (alindsey@umn.edu)
[1]Department of Entomology, University of Minnesota, St. Paul, Minnesota, 55108

Supplemental Figures

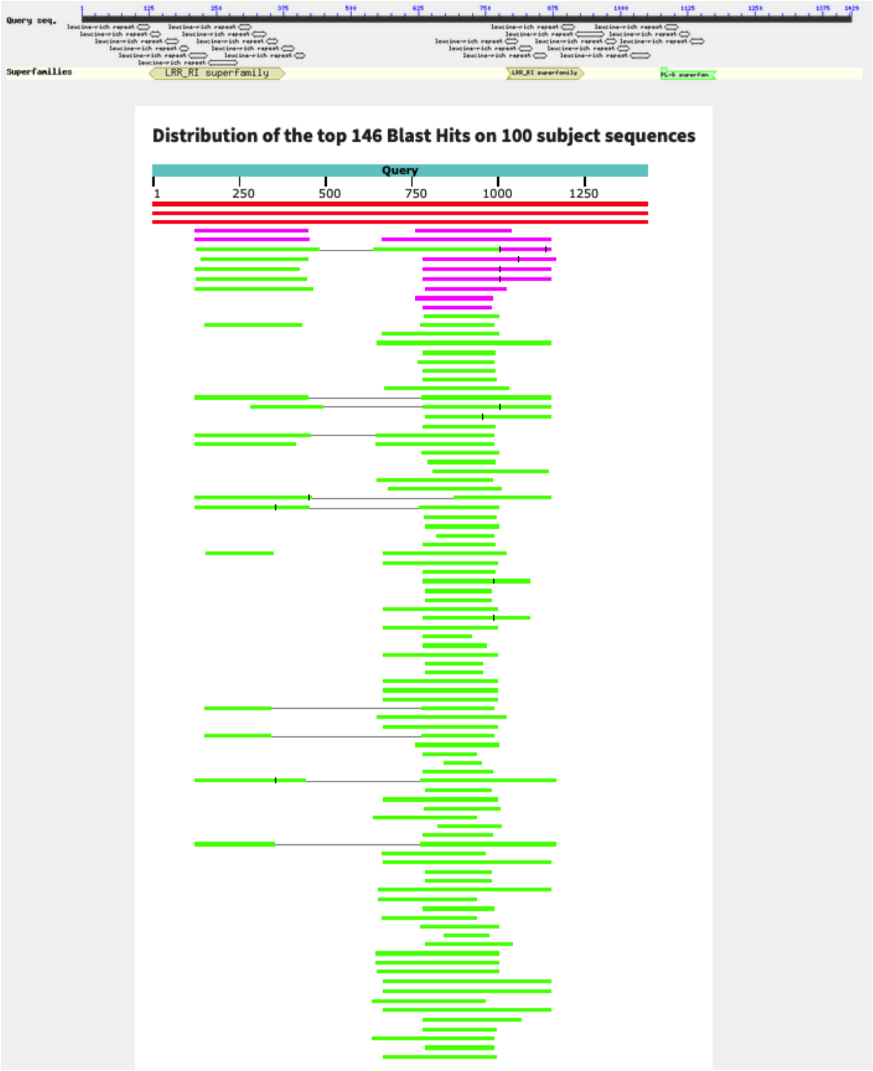

Figure S1. PifB BLASTP matches outside of *w*Tpre and *w*Lcla are restricted to low similarity LRR-like domains in other proteins.

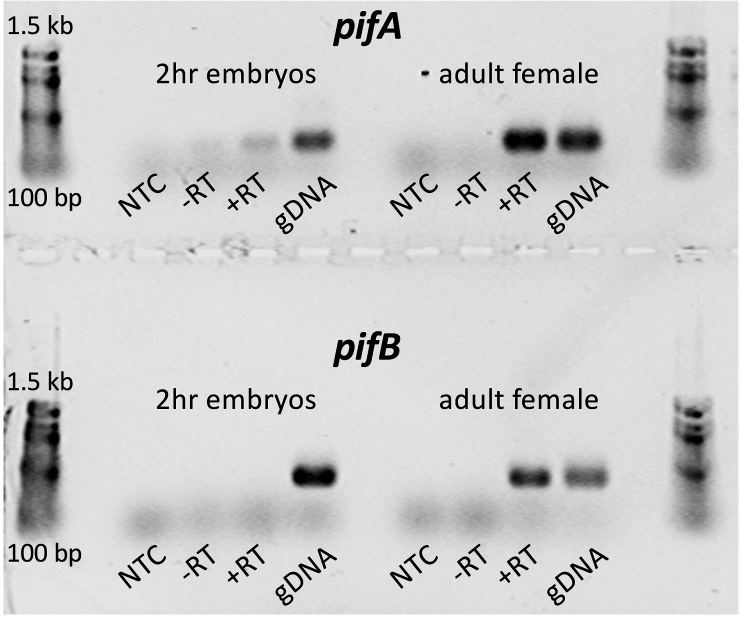

Figure S2. *pifA* and *pifB* are expressed in adult female *Trichogramma pretiosum*. RT-PCR was used to amplify ~300 bp regions of *pifA* and *pifB* from two hour old embryos, and adult females. Controls include no template (NTC) and no reverse transcription (-RT) negative controls, and a gDNA positive control.

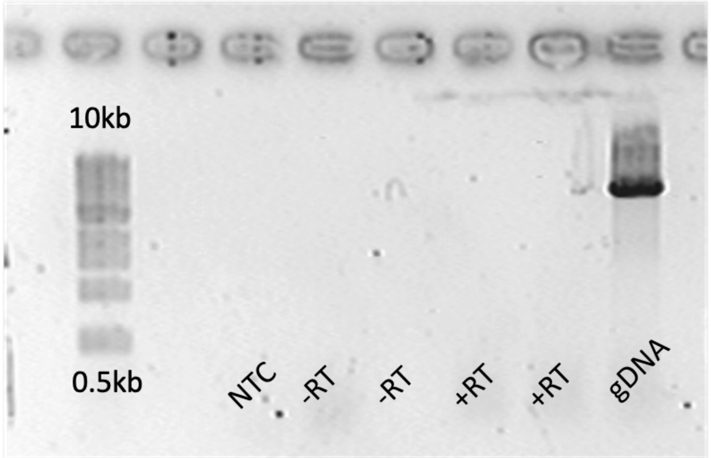

Figure S3. *pifA* and *pifB* are not cotranscribed in *Trichogramma pretiosum.* RT-PCR was used to amplify across *pifA* and *pifB* cDNA from adult females. Controls include no template (NTC) and no reverse transcription (-RT) negative controls, and a gDNA positive control.

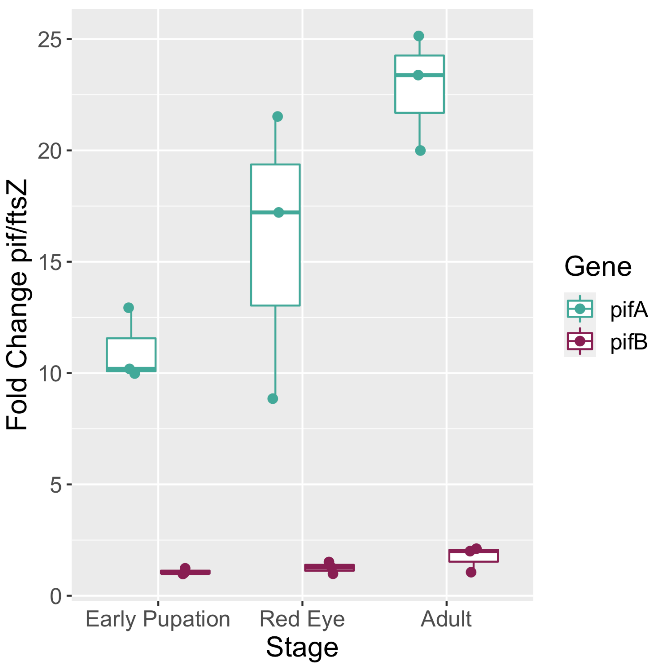

Figure S4. *w*LcIa pif expression increases across wasp development
qRT-PCR was used to quantify expression of *pif* loci across development, and was normalized to *ftsZ*. *pif* expression increases through wasp development for both *pifA* and *pifB*. There was no expression present in the early embryo.
